# Long-Acting Injectable Antiretroviral Therapy as a Catalyst for Engagement in Opioid Use Disorder Treatment Among People With HIV Who Use Drugs

**DOI:** 10.1093/ofid/ofag689

**Published:** 2026-10-24

**Authors:** Elizabeth Whidden, Amanda Perez, M Elle Saine, Jessica Meisner

**Affiliations:** Department of Medicine, Perelman School of Medicine, University of Pennsylvania, Philadelphia, Pennsylvania, USA; Department of Medicine, Perelman School of Medicine, University of Pennsylvania, Philadelphia, Pennsylvania, USA; Department of Medicine, Perelman School of Medicine, University of Pennsylvania, Philadelphia, Pennsylvania, USA; Leonard Davis Institute of Health Economics, University of Pennsylvania, Philadelphia, Pennsylvania, USA; Department of Medicine, Perelman School of Medicine, University of Pennsylvania, Philadelphia, Pennsylvania, USA; Prevention Point Philadelphia, Philadelphia, Pennsylvania, USA

**Keywords:** HIV, long-acting injectable treatment, medication for opioid use disorder, treatment engagement

## Abstract

**Background:**

People with substance use disorders (SUDs) living with human immunodeficiency virus (HIV) experience overlapping structural and clinical barriers to care. Long-acting injectable antiretroviral therapy (LAI-ART) may improve treatment adherence and may strengthen engagement in health services. Whether initiation of LAI-ART is associated with subsequent uptake of medications for opioid use disorder (MOUD) is unknown.

**Methods:**

We conducted a retrospective descriptive cohort study of people with HIV and a history of a SUD who initiated long-acting cabotegravir-rilpivirine (LA-CAB + RPV) at a low-barrier social services and public health organization between February 1, 2022 and June 30, 2025 and received at least 6 months of follow-up. We evaluated whether initiation of this regimen improved HIV and SUD outcomes, including uptake of MOUD, sustained treatment engagement, reduced opioid use, and viral suppression.

**Results:**

Forty patients initiated LA-CAB + RPV. Viral suppression improved from 73% at baseline to 98% at follow-up. Adherence was high, with only one missed scheduled dose and no recorded deaths from HIV-related causes. At baseline, 81.1% reported active opioid use and 32.4% were receiving medications for OUD. MOUD utilization increased to 78.4% at 6 months and 89.2% at end follow-up, while opioid use declined to 59.5% and 48.6%. Among those initiating medications for OUD after CAB + RPV, 90.9% remained on treatment, with 40% initiating within 2 months. Most patients remained engaged in HIV care.

**Conclusions:**

Integrated low-barrier care models may improve continuity of care for marginalized populations.

## Teaser

Among people with HIV and substance use disorder, initiating long-acting cabotegravir-rilpivirine in a low-barrier clinic significantly improved viral suppression, increased medication for opioid use disorder uptake, reduced opioid use, and maintained high treatment engagement over 6 months.

The syndemic of human immunodeficiency virus (HIV) and opioid use disorder (OUD) remains a persistent driver of morbidity and mortality in the United States [1]. People with a substance use disorder (SUD) bear a disproportionate burden of HIV acquisition and transmission due to intersecting structural and behavioral factors, including syringe sharing, housing instability, criminalization, incarceration, and limited access to consistent, longitudinal medical care [2, 3]. Despite major advances in antiretroviral therapy (ART), people living with HIV (PLWH) who use drugs continue to experience lower rates of retention in care and viral suppression compared with people without SUDs [3, 4].

OUD is a chronic medical condition associated with high all-cause mortality, driven largely by overdose risk [5]. Evidence-based medications for opioid use disorder (MOUD)—primarily buprenorphine and methadone—substantially reduce all-cause mortality [6, 7]. However, MOUD remains markedly underutilized, particularly among people with OUD who have been socially and economically marginalized [8, 9]. As a result, many individuals with HIV and OUD do not receive effective, comprehensive treatment. This perpetuates excess morbidity and mortality and limits the real-world efficacy of these evidence-based therapies.

Philadelphia is a key setting in which to develop and implement novel approaches to address the syndemics of OUD and chronic viral infections, such as HIV. The city has one of the highest overdose mortality rates among large US cities and a substantial population of individuals living with both HIV and OUD [10–13]. Many experience unstable housing, criminalization of substance use including incarceration, and frequent disruptions to care [14, 15]. These overlapping societal challenges make patients vulnerable to poor outcomes and highlight the need for integrated, low-barrier care models that address HIV and OUD simultaneously rather than as siloed conditions.

Long-acting injectable antiretroviral therapy (LA-ART) represents a paradigm shift in HIV treatment delivery [16]. Cabotegravir plus rilpivirine (CAB + RPV), a LA-ART regimen approved in the US in 2021, eliminates the need for daily oral dosing and has demonstrated noninferior viral suppression compared with oral ART in clinical trials [16]. Emerging real-world evidence suggests that LA-ART may be particularly beneficial for individuals facing barriers to daily adherence, including those with housing instability, incarceration, and active substance use [17]. Prior implementation studies, including our earlier case series from a clinic embedded within a syringe service program, have demonstrated high rates of viral suppression, adherence, and retention in care among PWID receiving CAB + RPV in low-barrier settings [18, 19].

Beyond virologic outcomes, LA-ART may influence broader patterns of healthcare engagement, particularly for people with co-occurring SUD. While LA-ART's impact on HIV treatment adherence is well-documented, less is known about whether its structural advantages translate to improvements in managing concurrent conditions. Regularly scheduled injection visits create predictable and regular points of contact with the healthcare system every 2 months, and reduced treatment burden may enhance self-efficacy and trust among patients who have previously experienced difficulty taking daily medication and/or stigma. We hypothesize that the low-barrier nature of LA-ART delivery creates stable touchpoints with the healthcare system, and increased ability to manage HIV improves patients’ self-efficacy. By simplifying HIV treatment and increasing continuity of care, LA-ART may also affect engagement with treatment for co-occurring conditions that share similar barriers to access and retention.

To our knowledge, no prior studies have explicitly examined whether initiation of LA-ART facilitates subsequent uptake and sustained engagement in MOUD among people with SUD. We previously reported on our implementation work at a syringe services program-embedded clinic [18] in this follow-up study, we evaluate whether initiation of CAB + RPV is associated with improved HIV and SUD outcomes—such as increased MOUD uptake, sustained engagement in OUD treatment, reductions in opioid use, and long-term viral suppression among people with SUD and people with HIV receiving care in a low-barrier clinical setting.

## METHODS

### Study Design and Setting

We conducted a retrospective descriptive cohort study using electronic health record (EHR) data from the Sana Clinic. The Sana Clinic is a low-barrier HIV primary care clinic embedded within Prevention Point Philadelphia, a social services and public health organization operating a large syringe service program in the Kensington neighborhood of Philadelphia. It provides comprehensive care to PLWH, including LA-ART, primary care services, and MOUD.

Patients were followed through December 31, 2025. A 6-month postinitiation time point was included as a standardized evaluation window to ensure sufficient time to evaluate patterns of care access.

### Study Population

Patients were included if they were (1) adults aged >18 years, (2) had a documented diagnosis of HIV, (3) initiated CAB + RPV between February 1, 2022 and June 30, 2025, (4) had a documented history of SUD, and (5) received at least 6 months of CAB + RPV. Patients were followed through December 31, 2025.

### Data Collection

EHRs were reviewed for all patients who met inclusion criteria. A trained abstractor (E.W.) performed manual chart abstractions into structured forms through Research Electronic Data Capture (REDCap), a secure web application for building and managing online surveys and databases [20]. Extracted variables included demographic characteristics (age, race, ethnicity, and gender), housing status, substance use patterns (opioid and nonopioid substances), HIV treatment history, CAB + RPV dosing and missed injections, HIV viral load measurements, MOUD prescriptions, timing of MOUD initiation relative to CAB + RPV start, retention in care, and mortality. All data were deidentified and stored securely in REdCap.

### Study Design

Baseline was defined as the most recent clinical encounter prior to initiation of CAB + RPV. Baseline opioid use was defined as documented ongoing nonprescribed opioid use based on clinician assessment and patient self-report at that encounter. Baseline MOUD use was defined as consistent receipt of prescribed buprenorphine (at least 7 weeks of oral buprenorphine or 2 doses of injectable buprenorphine received in the 8 weeks prior to CAB + RPV initiation). This was abstracted from the medication dispense report in the EHR or documented receipt of MOUD from an external program.

Rates of HIV suppression (<200 copies/mL) at baseline, at 6 months, and at the end of follow-up are reported. Patterns of adherence—including number of late CAB + RPV (injection >2 weeks after scheduled due date) and missed CAB + RPV doses (injection >4 weeks after scheduled due date)—were evaluated. We additionally assessed how often patients received doses at outside facilities, including hospitals, rehab centers, and correctional centers. Retention in care and all-cause mortality at the end of follow-up are reported. Retention on MOUD was defined at each assessment time point (baseline, 6 months post-CAB + RPV initiation, and end of follow-up) as at least 7 weeks of oral buprenorphine or 2 doses of injectable buprenorphine received during the 8-week period immediately preceding that time point.

### Outcomes

Descriptive analyses were conducted for the full cohort of 40 patients with HIV and SUD to characterize baseline demographics, HIV treatment outcomes, CAB + RPV adherence, and retention in care. Among these, we identified a subgroup of patients with a history of OUD, defined as patient self-report and/or clinician documentation in the medical record indicating current or prior OUD. Statistical analyses of MOUD-related outcomes were restricted to this OUD subgroup. Primary outcomes were both MOUD use and opioid use at 6 months following CAB + RPV initiation and at the end of follow-up (December 31, 2025).

### Statistical Analysis

Demographic and clinical characteristics were summarized descriptively as counts (with frequencies), and mean or median (with standard deviation or interquartile range), as appropriate. Paired comparisons of MOUD use and opioid use at time of CAB + RPV initiation compared at 6-months after initiation and at the end of follow-up were conducted using McNemar's tests. All analyses were performed using Stata 19.0 (Stata Corporation, College Station, TX).

### Ethics

This study was reviewed by the Institutional Review Board of the Philadelphia Department of Public Health. A waiver of informed consent and HIPAA authorization was granted, as all data were deidentified and collected retrospectively.

## RESULTS

### Patient Baseline Characteristics

Between February 1, 2022 and June 30, 2025, 40 patients with HIV and SUD were initiated on CAB + RPV and received at least 6 months of treatment. Most patients were aged 30–39 years (50.0%) or 40–49 years (30.0%) (Table 1). The majority identified as male (65%) and cisgender (95%). The cohort was racially and ethnically diverse: 57.5% identified as White, 25% as Black/African American, and 17.5% as Hispanic/Latinx.

**Table 1.** Baseline Characteristics of All Patients Initiated on Long-Acting CAB + RPV (N = 40)

Characteristic	Total (N = 40)	With OUD (N = 37)
Age in years (n%)		
20–39	21 (52.5%)	19 (54%)
40–49	12 (30%)	8 (21.6%)
> 50	7 (17.5%)	9 (24.3%)
Gender identity (n%)		
Male	26 (65%)	24 (59%)
Female	14 (35%)	13 (41%)
Identify as cisgender	38 (95%)	36 (97%)
Race/ethnicity (n%)		
Black/African American	10 (25%)	9 (24%)
White	23 (57.5%)	22 (59%)
Hispanic/Latinx	7 (17.5%)	6 (16%)
Housing status (n%)		
Street unhoused	15 (37%)	15 (41%)
Shelter	8 (20%)	8 (22%)
Stably housed	17 (43%)	14 (38%)
Active substance use at CAB + RPV initiation (n%)		
Opioid use	30 (75%)	30 (81%)
Stimulant use	27 (68%)	26 (70%)
On MOUD at CAB + RPV initiation (n%)	12 (32%)	12 (32%)
Oral buprenorphine	3 (7.5%)	3 (8%)
Injectable buprenorphine	8 (20%)	8 (22%)
Methadone	1 (2.5%)	1 (2.7%)
HIV viral suppression at baseline (n%)		
HIV RNA <200	29 (72.5%)	26 (67.6%)
HIV RNA 201–1000	5 (12.5%)	5 (13.5%)
HIV RNA 1001–5000	4 (10%)	4 (10.8%)
HIV RNA 5001–10 000	0	0
HIV RNA 10 001–20 000	1 (2.5%)	1 (2.7%)
HIV RNA >20 000	1 (2.5%)	1 (2.7%)
Most recent HCV serology Ab+	34 (85%)	32 (86%)
Most recent Heb B serology		
Immune from prior exposure	18 (45%)	17 (46%)
Immune from vaccination	22 (55%)	20 (54%)

Only 43% of patients were stably housed, while 20% were living in shelters and 37% were unsheltered and staying on the street at the time of CAB + RPV initiation.

At the time of CAB + RPV initiation, 72.5% of the cohort had HIV viral suppression (HIV RNA <200 copies/mL). Among those with detectable viremia, 12.5% had HIV RNA 201–1000 copies/mL, 10% had 1001–5000 copies/mL, 2.5% had 10 001–20 000 copies/mL, and 2.5% had HIV RNA >20 000 copies/mL.

Most patients were hepatitis C antibody positive (85%). All patients had positive hepatitis B surface antibody prior to CAB + RPV initiation; 45% had evidence of prior hepatitis B exposure (core antibody positive and surface antigen negative), and 55% had immunity from vaccination.

The majority (92.5%) of patients had a history of OUD. Of these 37, 30 (81.1%) patients reported active opioid use and 12 (32%) were already receiving MOUD at the time of CAB + RPV initiation. Eight were prescribed long-acting injectable buprenophrine (LAIB), 3 were prescribed sublingual buprenorphine, and 1 was on methadone. Baseline characteristics of the cohort with OUD are reflective of the larger cohort described above.

### CAB + RPV Adherence and Retention

HIV viral suppression improved substantially over time (Table 2). At the end of follow-up, 39 of 40 patients (97.5%) achieved HIV RNA <200 copies/mL; 1 patient (2.5%) had HIV RNA 201–1000 copies/mL. Among patients with OUD, 97.3% were suppressed at the end of follow-up.

**Table 2.** HIV Outcomes and Retention in Care

Outcome	Total (N = 40)	Group With OUD (N = 37)
HIV viral suppression at 6 m from CAB + RPV initiation (n%)		
HIV RNA <200	39 (97.5%)	336 (97.3%)
HIV RNA 201–1000	1 (2.5%)	1 (2.7%)
HIV viral suppression at end of follow-up (n%)		
HIV RNA <200	39 (97.5%)	36 (97.3%)
HIV RNA 201–1000	1 (2.5%)	1 (2.7%)
Patients with missed CAB + RPV doses	1 (2.5%)	0 (0%)
Patients with CAB + RPV doses >2 wks late	2 (5%)	2 (5.5%)
Patients who received doses at another site	13 (32.5%)	12 (32.4%)
Patients who received a dose while incarcerated	9 (22.5%)	9 (24.3%)
Of those living, retained in care at end of follow-up	35 (97.2%)	33 (100%)
All-cause mortality	4 (10%)	4 (10.8%)

Adherence to CAB + RPV injections was high. Only 1 patient (2.5%) missed a scheduled dose (>4 weeks late), and 2 patients (5%) received doses more than 2 weeks late.

Continuity of care was maintained across care settings. Thirteen patients (32.5%) received at least 1 CAB + RPV dose at an alternative site, including hospitals, treatment centers, and correctional settings. Nine patients (22.5%) received at least 1 dose while incarcerated.

Among the 36 patients alive at the end of follow-up, 35 (97.2%) were retained in care. Four patients (10%) died during the follow-up period, all of whom were virally suppressed. We did not collect more granular information on cause of death.

### Outcome: Changes in Opioid Use and MOUD Use Following CAB + RPV Initiation

Among patients with OUD (N = 37), CAB + RPV initiation was associated with increased MOUD uptake and decreased opioid use over time (Table 3).

**Table 3.** Changes in Opioid Use and MOUD Use Following CAB + RPV Initiation for People With a History of Opioid Use Disorder (N = 37)

Outcome	Baseline	6 M	End of Follow-up
Active opioid use, n (%)	30 (81.1%)	22 (59.5%)^a^	18 (48.6%)^a^
Receiving MOUD, n (%)	12 (32.4%)	29 (78.4%)^a^	33 (89.2%)^a^
Receiving LAIB, n (%)	8 (21.6%)	15 (40.5%)^a^	21 (56.7%)^a^

^a^Signifies that *P*-values of time point vs baseline are <.01.

MOUD use increased from 32.4% at baseline to 78.4% at 6 months (*P* < .001), and to 89.2% at end of follow-up (*P* < .01) (Figure 1). Only 3 patients (11.8%) did not initiate MOUD during the follow-up period. Active opioid use declined from 81.1% at baseline to 59.5% at 6 months (*P* < .01), and further to 48.6% at end of follow-up (*P* < .01).

**Figure 1.**
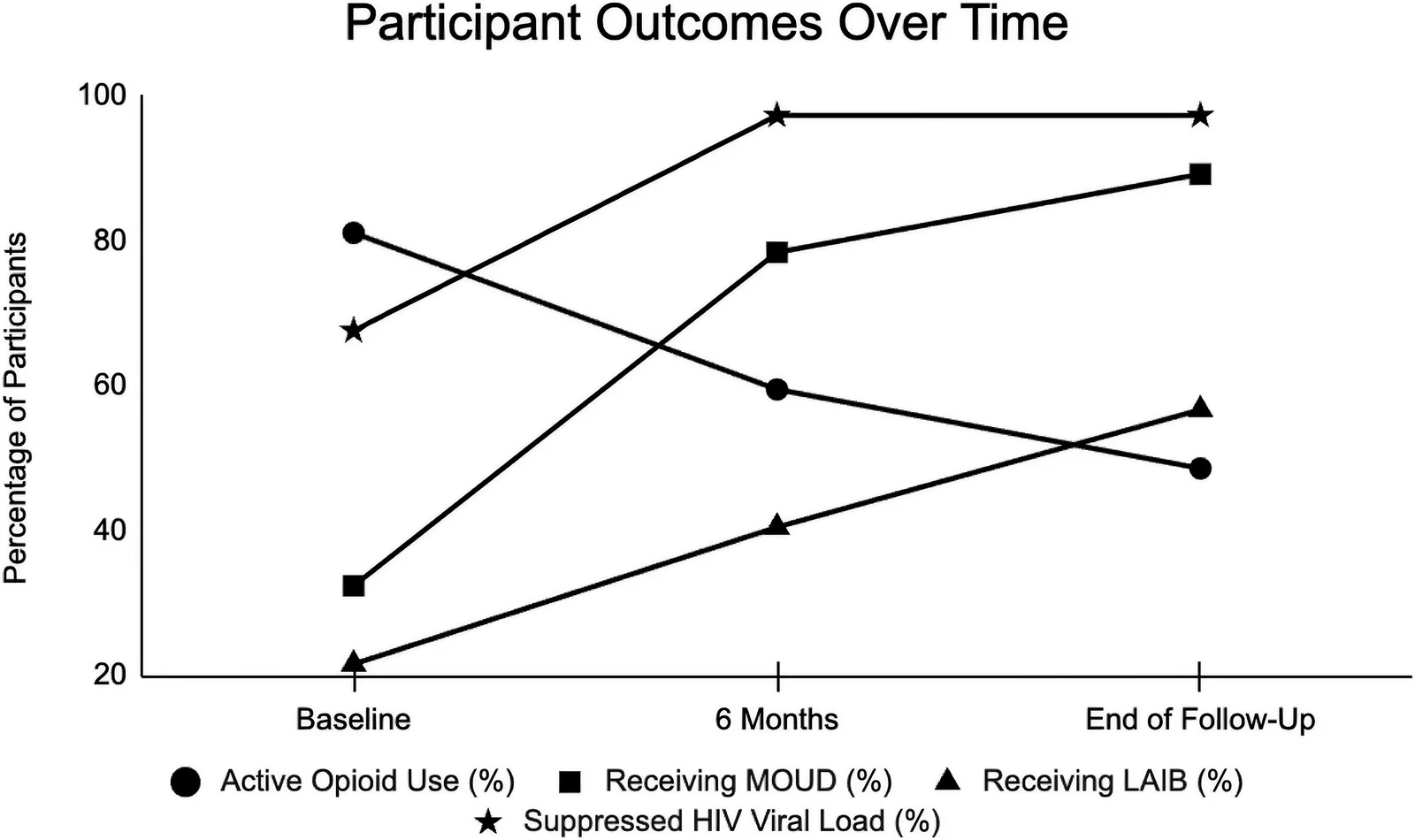
Changes in Opioid Use and MOUD Use over time following CAB + RPV Initiation for people with a history of opioid use disorder (N = 37).

Use of LAIB also increased over time. At baseline, 21.6% of patients were receiving LAIB, increasing to 40.5% at 6 months (*P* < .01) and 56.7% at the end of follow-up (*P* < .01).

MOUD initiation occurred early—10 patients (40%) initiated within 0–2 months, 5 (20%) within 3–6 months, and 7 (28%) after 6 months (Table 4). Of these 22 patients who initiated MOUD during the study period, 20 (90.9%) remained on it at the end of follow-up.

**Table 4.** Timing and Retention of MOUD Initiation After CAB + RPV for People With a History of Opioid Use Disorder and no MOUD at Baseline (N = 25)

MOUD Outcome	Total (N = 25)
Initiated MOUD after CAB + RPV	22 (88%)
Never initiated MOUD after CAB + RPV	3 (12%)
Timing of MOUD initiation	
Within 0–2 m	10 (40%)
Within 2–6 m	5 (20%)
> 6 m	7 (28%)
Of those who initiated MOUD in window, were retained on MOUD at end of follow-up	20/22 (91%)

## DISCUSSION

In this retrospective study of PLWH and iSUD receiving care in a low-barrier clinic, initiation of CAB + RPV was associated with excellent HIV outcomes alongside substantial improvements in engagement with MOUD among the subset of patients with OUD. Viral suppression increased from approximately three-quarters of patients at baseline to near-universal suppression by the end of follow-up, with high adherence to CAB + RPV and retention in care among surviving patients. In parallel, initiation of CAB + RPV was associated with rapid and sustained increases in uptake of MOUD as well as significant reductions in reported opioid use. Among patients with OUD who were not receiving MOUD at initiation of CAB + RPV, the vast majority initiated MOUD during follow-up, and nearly all who initiated remained engaged through the end of follow-up. Together, these findings suggest that LA-ART initiation may serve as a powerful entry point for engagement with OUD treatment in a population that has historically faced substantial barriers to both HIV and addiction care.

The near-universal viral suppression observed in our cohort is consistent with emerging evidence that LA-ART can lead to excellent virologic outcomes even among populations facing significant adherence challenges. Prior clinical trial and real-world studies have demonstrated the effectiveness of LA-ART among people with adherence difficulties and people who use drugs, including in settings serving patients with significant barriers to consistent care (Rana, Hickey). Our findings extend this evidence to a low-barrier clinic setting serving a highly marginalized population with substantial housing instability and active substance use, populations often excluded from clinical trials.

The magnitude and durability of MOUD uptake observed in this cohort are particularly striking given the high prevalence of housing instability, ongoing substance use, and disruption in care due to hospitalizations and incarcerations. At baseline, fewer than one-third of patients with OUD were receiving MOUD, mirroring national patterns. Following CAB + RPV initiation, MOUD use increased nearly 3-fold within 6 months and continued to rise through the end of follow-up. This increase was accompanied by a meaningful decline in active opioid use over time. Prior work has shown how patients on MOUD are 4 times more likely to start ART, 3 times more likely to adhere, and twice as likely to achieve viral suppression (Juwono). Our findings suggest that the converse may also be true and support the hypothesis that effective HIV treatment strategies may positively influence engagement with treatment for co-occurring conditions that share similar structural and psychosocial barriers.

The timing of MOUD initiation further underscores the potential role of LA-ART as a catalyst for broader healthcare engagement. Nearly half of patients initiated MOUD within 2 months of starting CAB + RPV, and more than 70% did so within 6 months, indicating that the period immediately following CAB + RPV initiation may represent a critical window of increased readiness or capacity to engage in OUD treatment. Importantly, MOUD engagement was sustained once initiated, with over 90% of patients remaining on treatment through the end of follow-up. The increasing use of LAIB over time may have further contributed to this durability, as long-acting formulations reduce the risk of treatment interruptions and provide ongoing overdose protection during periods of instability. These findings highlight the importance of leveraging HIV care to improve addiction-related outcomes and reduce rates of overdose. Further efforts are needed to close the research and recommendation gap to practice.

Taken together, these results reinforce the growing recognition that engagement in HIV care and addiction treatment is mutually reinforcing rather than competing priorities. Our findings suggest that achieving effective, low-barrier HIV treatment through LA-ART may reduce barriers that impede engagement with OUD treatment. Several mechanisms may underlie these observed associations. LA-ART eliminates the need for daily oral dosing, reducing a major barrier for patients experiencing housing instability or chaotic daily routines. Regularly scheduled injection visits create predictable, low-threshold points of contact with the healthcare system, strengthening continuity of care and therapeutic relationships. Early virologic success with CAB + RPV may provide tangible reinforcement of treatment efficacy, increasing motivation to address other health needs. Over time, sustained HIV suppression combined with engagement in MOUD may interrupt cycles of acute care utilization, unmanaged substance use, and overdose risk, leading to more stable long-term outcomes. The concurrent reductions in opioid use and high retention on MOUD observed in this cohort suggest that LA-ART may increase engagement in OUD treatment by normalizing ongoing engagement with care in a supportive, nonpunitive environment.

Overall, these findings support integrated, low-barrier care models that treat HIV and OUD simultaneously rather than siloed conditions. In settings with high overdose mortality and unmet need for MOUD, initiation of LA-ART may represent a strategic opportunity to strengthen engagement across both treatment cascades. The observed temporal pattern—rapid MOUD uptake following CAB + RPV initiation with concurrent reductions in opioid use—suggests that LA-ART may serve as a key intervention point for enhancing engagement in substance use treatment.

This study has limitations. Its retrospective design limits causal inference, and the modest sample size from a single clinic may limit generalizability. Continuity of CAB + RPV across hospitalizations and incarcerations was made possible through significant efforts from the care team, which may not be possible at other similar organizations. Substance use outcomes also relied on clinical documentation and self-report, and patients who initiated CAB + RPV may differ in unmeasured ways from those who declined. Nonetheless, the high prevalence of housing instability, active substance use, and prior adherence challenges underscores the relevance of these findings to highly marginalized populations.

In conclusion, among people with HIV and a history of injection drug use, CAB + RPV initiation was associated with increased uptake and sustained engagement in MOUD as well as with excellent HIV outcomes, suggesting that simplified HIV treatment through LA-ART may support broader engagement in addiction care.
